# Integrating time in definitions of carbon sequestration and greenhouse gas removals and reversals

**DOI:** 10.1098/rsos.242095

**Published:** 2025-06-04

**Authors:** Carlos Sierra

**Affiliations:** ^1^ Max Planck Institute for Biogeochemistry, Jena, Germany

**Keywords:** climate change mitigation, nature-based solutions, carbon dioxide removal, compartmental systems, Paris Agreement Crediting Mechanism, carbon cycle

## Abstract

The permanence or temporary nature of carbon removals from the atmosphere into natural systems has been a major topic of debate in the design of accounting methodologies for climate policy. These topics are currently being discussed in the preparation of a new mechanism established by the Paris Agreement. Emerging concepts from the field of ecosystem ecology could provide the key metrics needed to address the problems of permanence and reversibility of removals in carbon accounting. I show here how the concept of compartmental system provides the level of generality needed to address the temporal nature of removals in carbon accounting, with clearly defined metrics for assessing climate benefits of temporary storage. I also show how the variable time can be incorporated in the definitions of carbon sequestration and removals in a way that would encourage the development of climate actions that would keep carbon out of the atmosphere for long periods of time. In contrast to traditional ‘stock change’ or ‘tonne-year’ accounting methods, the approach proposed here takes explicit consideration of the time that carbon remains stored in a natural system and does not contribute to warming in the atmosphere. The proposed methodology may resolve current issues related to the definition of baselines, permanence and quantification of temporary storage and reversals.

## 1. Introduction

Article 6, paragraph 4 of the Paris Agreement created a mechanism to promote the mitigation of greenhouse gas (GHG) emissions and to incentivize and facilitate cooperation among countries through transfers of carbon credits. This mechanism, now called the Paris Agreement Crediting Mechanism (PACM) will supersede the Clean Development Mechanism (CDM) of the Kyoto Protocol and will provide the legal framework for the development of projects that would remove GHG from the atmosphere, storing carbon in natural or man-made reservoirs for a period of time. The supervisory body in charge of the implementation of this mechanism is currently working on the definitions and methodological standards to be adopted by the parties of the Paris Agreement.

One contentious issue in the definition of GHG removals is how to integrate the time that a GHG removed from the atmosphere would remain stored in a particular system and how to assign credits due to mitigation of climate change over a particular period of time given that carbon is not indefinitely stored in most natural systems. This discussion is particularly relevant for the design of nature-based solutions that remove carbon dioxide from the atmosphere in agriculture, forestry and conservation, and it is also now relevant for technological approaches of carbon dioxide removal. During the design and implementation of the CDM, there were already important discussions about how to deal with the permanence of carbon in a proposed project, how to account for the time carbon remains stored in a natural reservoir and how to make an equivalence between emissions and removals of GHGs [1,2]. ‘Tonne-year’ accounting emerged from these previous discussions, which is a set of methodologies that attempt to make an equivalence between the integrated area of an emission pulse over a time horizon and the amount of carbon that should be stored in a system to compensate for this emission. Those discussions and tonne-year accounting are resurfacing now under the design of article 6.4 mechanism [3–5], but in comparison with those previous discussions, there have been important conceptual advances in carbon cycle science and biogeochemistry that can shed some light now on designing the legal framework for defining GHG removals under the PACM. In fact, a new set of metrics that improve over tonne-year accounting and better account for the time of carbon storage have been recently proposed in the scientific literature [6–9].

Although the methods commonly known as ‘tonne-year’ accounting partially address the problem of temporary carbon storage, they also have serious flaws that need to be addressed before they are incorporated into a carbon trading scheme such as the PACM [3,10–12]. These methods create a false equivalence between the time that a pulse emission of CO_2_ spends in the atmosphere versus the amount of carbon that should be stored on land to compensate that emission. I will explain this in more detail in §7 and will concentrate first on explaining why accounting for the time that carbon spends stored in a particular system on land (not in the atmosphere) is essential for an appropriate accounting system of carbon removals and reversals.

The aim of this article is to summarize recent scientific progress on the quantification of timescales of carbon removals and emissions using the framework of compartmental dynamical systems and showing how these concepts could be adopted in the design of the PACM. This framework offers a high level of generality that could be used to account not only for the amount of carbon that can be stored in a system but also for the time it remains out of the atmosphere and does not contribute to radiative forcing. I will first introduce the concept of compartmental system, distinguish between gross and net fluxes and show how to integrate time in the quantification of removals. I will present a definition of carbon sequestration (CS) that, compared with a previously presented set of formulae [9], is much simpler to compute and does not require the explicit calculation of the transit time of carbon.

## 2. Storing carbon in a compartmental system

**Figure 1.**
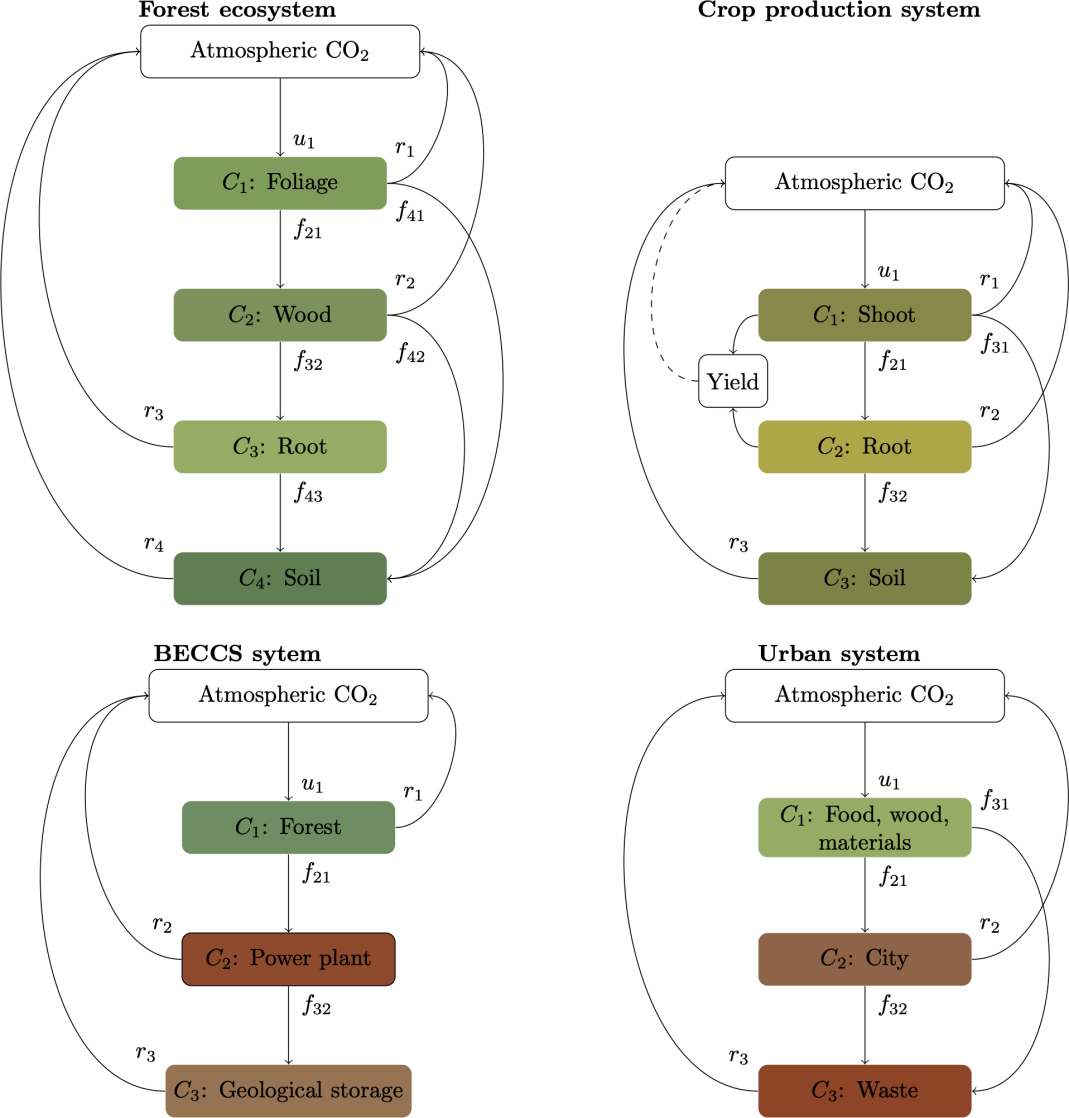
Examples of different systems that store carbon and exchange it with the atmosphere and that can be expressed as a compartmental system. Coloured boxes represent compartments that store carbon and that are included in an accounting system. Empty boxes are components of the system that do not need to be included in the accounting system. The number of compartments can be different for any system, and the total amount of carbon at any given time would be equal to the sum of carbon in all compartments. Each system has gross removals from the atmosphere defined by the symbol u, and gross reversal fluxes defined by the symbol r. The net flux, defined in the text as n, is the difference between all removals and reversals at any given time. These fluxes are not necessarily constant, and catastrophic events such as a forest fire can be quantified by increasing the transfer of carbon from one or multiple compartments to the atmosphere at any given time. For details about the symbols and how to build a compartmental system from these stocks and fluxes, see appendix A. BECCS: bioenergy with carbon capture and storage.

Important scientific progress in carbon cycle science and biogeochemistry has been achieved in recent years by studying carbon dynamics under the mathematical framework of compartmental dynamical systems [13–17]. A compartmental system is defined by one or many compartments that store carbon and may transfer carbon among them at specific rates (figure 1, appendix A). One can use this concept of compartmental system to represent natural systems such as soils, forests or crops, with internal compartments such as leaves, roots, microbes, etc. It can also be used to represent the balance of carbon in systems as diverse as a power plant, a city, a continent or the entire globe. The compartmental system contains carbon, and the change of carbon over time in the entire system can only occur due to changes in inputs or outputs of carbon. In other words, a compartmental system is always mass balanced.

One main advantage of this approach is that it changes the focus from specific process representation (which is still maintained in the internal equations that describe the dynamics of compartments) to a system-level analysis. For instance, this approach has facilitated progress in understanding the fate of photosynthetically fixed carbon, the time it spends in specific compartments and the time it requires to return to the atmosphere [13,18–20]. Of particular interest is the concept of transit time of carbon, which can be used to quantify how long carbon atoms need to pass through a compartmental system. For an ecosystem, the transit time of carbon can be defined as the time it takes carbon from the moment it enters via photosynthesis until it returns back to the atmosphere due to the respiratory activity of plants and soil microorganisms [18,19,21].

The transit time of carbon is a key concept to understand the temporary nature of carbon removals from the atmosphere. Not all activities that can remove carbon from the atmosphere would keep it stored for the same amount of time. In some systems, carbon may remain stored only for a few years or maybe decades, and in other systems it may remain stored for centuries or millennia. The concept of transit time explicitly quantifies this amount of time that certain amount of carbon removed from the atmosphere would remain stored in a particular system, and allows one to make comparisons among different systems or removal activities [7,22]. The quantification of transit time is greatly facilitated by studying reservoirs of carbon and biogeochemical elements as dynamical compartmental systems [13,23–25].

The permanence of carbon in single compartments or the entire system is an emerging property resulting from the pathways of carbon transfer and storage, it can be quantified by the transit time metric and does not need to be defined *a priori*; in other words, it is not necessary to create an *a priori* definition of permanence or long-term carbon storage as it is currently discussed in the development of the PACM.

## 3. Gross and net removals

Previous discussions in ecosystem science about gross and net fluxes of carbon in and out of ecosystems [26–29] have been fundamental for the development of current frameworks for carbon accounting in scientific investigations (e.g. Global Carbon Project (GCP) and Regional Carbon Cycle Assessment and Process (RECAPP-2), [30,31]). The change of carbon in a particular ecosystem (a compartmental system) can only occur due to the net balance between inputs and outputs of carbon. Definitions such as net ecosystem production, net ecosystem carbon balance or net biome production may differ regarding the scale of observation or the type of in and out fluxes being considered [29], but it is clear that the carbon balance in an ecosystem must be understood in the context of the net balance of carbon inputs and outputs. This net balance is exactly what a compartmental system aims at representing, and it serves as a template for representing the dynamics of carbon in other systems such as technological systems that remove CO${,}_{2}$ from the atmosphere and store it in underground facilities (e.g. bioenergy with carbon capture and storage) or other methods of direct carbon capture.

If we define u(t) as the gross uptake or removal flux of carbon from the atmosphere into a compartmental system at any given time t (units of mass divided by time) and r(t) as the gross release (reversal) flux from a compartmental system to the atmosphere (units of mass divided by time), then we can obtain the net removal flux as


(3.1) $$\begin{array}{lcr} & n(t)=u(t)-r(t), & \end{array}$$

where n(t) is the net removal flux, and it is similar to the concept of net ecosystem carbon balance at the ecosystem level or to net biome production at the landscape or continental level [29]. This net removal flux is also equivalent to the instantaneous change in carbon storage expressed as


(3.2) $$n(t)=\frac{dC(t)}{dt},$$

where C(t) is the total amount of carbon stored in the system at time t. Notice that neither n(t) nor C(t) tells something about the time that it takes to accumulate certain amount of carbon during a period of interest. C(t) only tells us how much carbon is stored in the system at the particular time t without any reference to whether that carbon was the result of a long period of accumulation, as in the case of an old-growth forest, or whether it is the result of a previous disturbance, and some amount of carbon was lost in comparison with a previous state.

If we agree on defining a previous initial time $t_{0}$, e.g. a pre-industrial reference year such as 1850, or a climate-policy reference year such as 1990 or the beginning of a project activity, we could integrate equation (3.2) as


(3.3) $$\int_{t_{0}}^{t}n(\tau )\;d\tau =C(t)-C(t_{0})$$

and obtain the carbon gain or loss from the system until time t with respect to the reference year $t_{0}$. The value of C(t) is reported in units of mass of carbon, potentially on an area basis (e.g. Mg C ha${,}^{-1}$). For convenience of notation, we can also refer to the result of equation (3.3) as


(3.4) $$\begin{array}{lcr} & \Delta_{t}C=C(t)-C(t_{0}), & \end{array}$$

where the subscript t indicates that the difference in carbon is with respect to carbon storage over time only (this subscript notation would become evident in §5). Equation (3.4) is commonly know as ‘stock change accounting’, and it is well known that this method of carbon accounting can introduce important biases [32].

If this difference in carbon stocks results in a positive change, then $\Delta_{t}C$ would be called CS by some authors (e.g. [33]), but this is a poor definition of CS because it does not account explicitly for the amount of time that carbon may be stored during the time interval under consideration [34]. It may be that the carbon stored remained for a short or for a long time stored in the system, and therefore an additional metric is required to integrate time.

**Figure 2.**
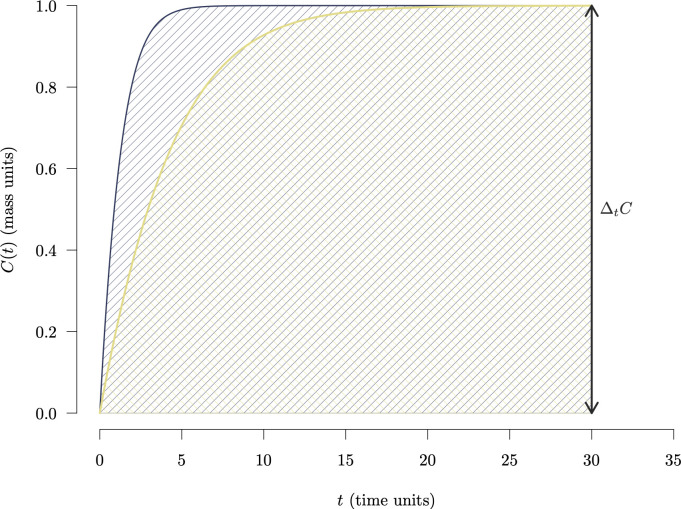
Trajectories of carbon stocks over time C(t) for two systems that differ in their rates of accumulation. At t=30, both systems have accumulated the same amount of carbon ($\Delta_{t}C=1.0$), but the faster system (darker colour) reached this maximum accumulation faster than the slower system (lighter colour). The value of $\Delta_{t}C$, which is the same for both systems, does not take into account their different temporal dynamics. However, the areas under the curve, represented by the their respective inclined lines, do take into account their differences. The faster system has a larger area under the curve, representing the fact that C has been stored for longer in this system.

As an example, consider two different systems that at some reference time $t_{0}=0$ contain no carbon, i.e. C(0)=0. This could be the case of an afforestation or reclamation project after open air mining in which we want to establish trees on a bare soil that contains no organic carbon, and we want to evaluate two different options of tree selection and forest management that lead to differences in the speed of carbon accumulation. After some time, say 30 units of time, both systems have accumulated the same amount of carbon. However, one of the systems reached this amount of carbon storage earlier than the other system (figure 2). If at t=30 one would compare both systems, they would be identical in terms of their carbon storage (C(t)) and their difference with respect to their initial carbon content ($\Delta_{t}C$). In the faster system, carbon is removed from the atmosphere for a longer amount of time, but the $\Delta_{t}C$ metric at t=30 does not account for this difference. However, comparing the areas under the C(t) curves, we can see that the faster system has an area that is 11% larger than the area of the slower system. The faster system has retained carbon out of the atmosphere for a longer period of time, and the combination of mass and time through the computation of an area provides a good metric for assessing how much and for how long carbon has been retained in these systems.

Ideally, a definition of CS, and an accounting methodology to quantify net removals of carbon from the atmosphere in the context of climate change mitigation and the PACM, should include both the amount of carbon stored in the system and the time it is kept stored and out of the atmosphere.

## 4. Carbon sequestration as time-integrated carbon storage

The example in figure 2 showed that stock changes ($\Delta_{t}C$) alone are not enough to tell apart temporal dynamics of carbon accumulation that would imply different amounts of time that carbon would be stored in a system. However, the areas under the curves in figure 2 do show these differences. Furthermore, if one would be interested in promoting carbon storage for longer times, one could extend the time frame of storage making these areas larger. This type of accounting would effectively address the time period that carbon is removed out of the atmosphere, and therefore it is a relevant metric for carbon accounting in climate change mitigation.

I define here CS as the storage of a certain mass of carbon (M) over a certain period of time ($t_{0}+T$) within a system, from an initial time $t_{0}$ over a time horizon T. It can be quantified as the area under the curve of a function that describes how this mass evolves over time (M(t))


(4.1) $$CS(t_{0},T):=\int_{t_{0}}^{t_{0}+T}M(t)\;dt;$$

because CS is an area, it is measured in units of mass multiplied by time [mass ⋅ time]. The notation CS$\left(t_{0},T\right)$ can be read as the amount of CS starting at an initial time $t_{0}$ over a time horizon T.

CS can be computed for two different cases: (i) considering only the inputs entering since $t_{0}$ without taking into consideration the stocks already present in the system and that may contribute to the release flux after $t_{0}$. A system may be empty with no carbon from a previous land use, as in the case of reclamation after mining; or it can have a legacy from a previous land use [35,36], but this carbon is not accounted under this case. (ii) Considering the legacy carbon present at $t_{0}$ that would contribute to future carbon losses and all further inputs until $t_{0}+T$.

### 4.1. Carbon sequestration considering only inputs during a time horizon

Depending on accounting methodologies and agreements among C market participants, it is possible to agree on an accounting methodology of C stocks that only takes into consideration the fate of new carbon inputs to the system during a specific time frame. This can occur in cases in which a project only takes responsibility for the new carbon entering the system under a proposed activity without any responsibility for the fate of legacy carbon that is not under the control of the project proponent. For example, a project activity on direct carbon capture and storage may only take responsibility of the CO${,}_{2}$ captured by the technological development but take no responsibility of the organic carbon present in the soil and plants surrounding the facilities and that may eventually release CO${,}_{2}$ to the atmosphere.

In this case, M(t) is determined only by the balance of the carbon inputs and outputs occurring in the system


(4.2) $$\begin{array}{lcr} & M_{u}(t)=\int_{t_{0}}^{t}u(\tau )-r_{u}(\tau )\;d\tau , & \end{array}$$

where τ is a ‘dummy variable’ of integration and is used only to avoid confusion with variable t.

In this case, the subscript u in the release rate $r_{u}(t)$ indicates that the losses from the system are only due to carbon that entered through u at an earlier time. This release rate $r_{u}(t)$ is easy to quantify for a system that was originally empty so no legacy carbon is present at $t_{0}$. However, if some carbon was already present in the system and contributes to the total release after $t_{0}$, a more complex computation needs to be done. In Sierra *et al*. [9], we present a detailed methodology for the case in which legacy carbon is excluded for the computations.

### 4.2. Carbon sequestration considering changes in total carbon stocks

Another possibility is that we are interested in accounting for both the fate of new carbon inputs and the fate of legacy carbon present in the system at $t_{0}$. This case is relatively simple to express mathematically because the mass of carbon under consideration is the net balance between the total inputs and outputs during the time horizon under consideration. Therefore,


(4.3) $$\begin{array}{rl} M(t) & =\int_{t_{0}}^{t}u(\tau )-r(\tau )\;d\tau \\ & =\int_{t_{0}}^{t}n(\tau )\;d\tau \\ & =C(t)-C(t_{0}). \end{array}$$

Then, CS in this case can be defined as


(4.4a)CS=∫t0t0+T∫t0tu(τ)−r(τ)dτdt(4.4b)=∫t0t0+T∫t0tn(τ)dτdt(4.4c)=∫t0t0+T(C(t)−C(t0))dt(4.4d)=∫t0t0+TC(t)dt−TC(t0).

We can see that CS can be computed in different ways: (i) integrating twice the net balance between carbon inputs and outputs (equations (4.4a) and (4.4b)), (ii) integrating the difference between initial carbon content and the curve of carbon contents over time (equation (4.4c)), and (iii) integrating only the curve of carbon contents over time and subtracting the product of the initial carbon content and the length of the time horizon (equation (4.4d)). The results obtained with any of these three forms should be identical, but one could apply any of them according to the type of information available.

Equation (4.4d) is useful to understand that CS does not integrate all the carbon stocks present in the system at the initial time. It only considers the stocks that accumulate or are lost during the period of interest. Therefore, the carbon contents at the initial time $C(t_{0})$ serve as a baseline in the computation of CS. If the system gains carbon during the period of interest, then CS is positive, and negative if the system loses carbon. If carbon in the system remains constant, then CS is zero, which implies that CS measures only the gains or losses of carbon that occur during the period of interest. If the system is empty at $t_{0}$, CS accounts for all the carbon accumulation occurring during the time horizon.

**Figure 3.**
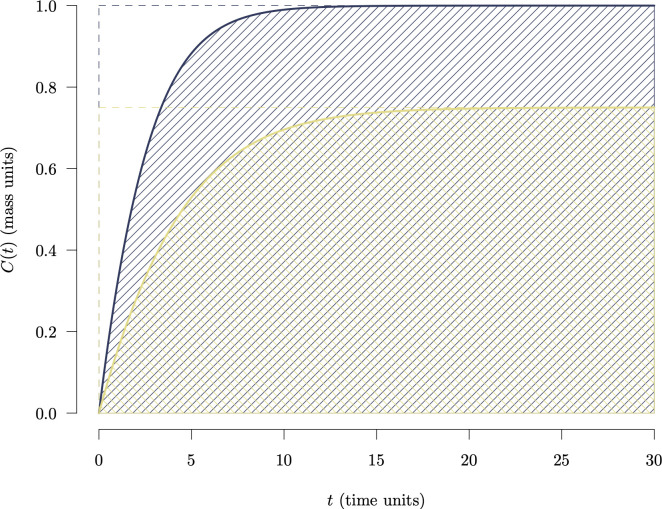
Trajectories of carbon stocks over time C(t) for two systems A (faster accumulation and larger stocks) and B (slower accumulation and lower stocks). System A contains 33% more carbon at t=30 than system B. However, in terms of area, system A has an area 41% larger than system B. The proportional difference in stocks at t=30 is similar as the proportional differences in the squared areas enclosed by the dashed lines. This implies that comparing by $\Delta_{t}C$ gives the same outcome as assuming that the two systems originally had the same amount of carbon since t=0.

As an example, consider two different systems that contain no carbon at the initial time (C(0)=0) (figure 3). System A accumulates carbon faster than system B and stores more carbon over a horizon of 30 time units. The difference in carbon contents at t=30 is 0.25 units of carbon. That is, system A stores at t=30, 33% more carbon than system B. If we compare their values of CS, i.e. the areas under the carbon stock curves, system A has an area that is 41% larger than system B. The difference with respect to area is proportionally larger because the areas take into account the time required for both systems to reach their final carbon content. Interestingly, if we would have assumed that both systems had an initial carbon content similar as their final carbon content (C(0)=C(30)), the proportional difference in the area would be 33%, a similar difference as when comparing based on stock change. In other words, on a proportional basis, comparing differences in the final stocks is similar to assuming that the carbon stock does not change over time, and the time that it takes for carbon to accumulate and get retained has no relevance.

Notice that CS does not give emphasis to either the gross or net removal flux, but rather to the amount of carbon and the period of time it has been stored. This is important in the context of carbon accounting in natural versus managed forests, where an emphasis on gross removal rates gives perverse incentives to cut down old-growth forests in favour of fast growing tree plantations [37]. The concept of CS avoids these perverse incentives and can promote the preservation of high stocks in old-growth forests for long periods of time (see also [7]).

## 5. Accounting with respect to a baseline

Accounting methodologies in carbon markets generally require the specification of a baseline, an estimate of the trajectory of carbon stocks in the absence of a project activity. Carbon stocks at the end of the project activity are generally compared with the estimated carbon stocks in the baseline, and their difference is what can be claimed as additional carbon credits.

This traditional accounting method does not take into consideration the time that carbon is kept out of the atmosphere. I argue that a more robust method for carbon accounting would take into account this time of carbon retention, using the conceptual approach of CS presented here. Instead of giving credits based on differences in carbon stocks with respect to a baseline, credits should be given based on a calculation of CS with respect to a baseline. This implies that the comparison should be done in units of [mass ⋅ time] instead of [mass].

The previous example showed that in the calculation of CS, the value of carbon stock at an initial time serves as a baseline. This is useful when there is not a different system or scenario to compare with, and we only have as a reference a previous carbon stock. For example, a national government may set as a goal to increase carbon stocks to a level above the carbon stocks present at some past reference year. In this case, the baseline is not the trajectory of a different system or scenario, it is simply the stocks present at some previous reference time. Therefore, changes in CS would assess whether there was a gain or loss of carbon-time during some commitment period.

Alternatively, the baseline can indeed be expressed in terms of a different system or scenario with a different carbon trajectory as the system of interest. In this case, it is useful to compare differences in carbon stocks or CS with respect to a baseline trajectory b. Therefore, we can define differences in terms of carbon storage at time t as


(5.1) $$\Delta_{b}C(t)=C(t)-C_{b}(t),$$

where C(t) would be the amount of carbon present in a particular system of interest and $C_{b}(t)$ would be the carbon storage of a baseline case. A similar metric can be defined for CS as


(5.2) $$\begin{array}{lcr} & \Delta_{b}\mathrm{CS}(t_{0},T)=\mathrm{CS}(t_{0},T)-{\mathrm{CS}}_{b}(t_{0},T). & \end{array}$$

While $\Delta_{b}C$ quantifies differences in [mass] among two systems, $\Delta_{b}$CS quantifies differences in [mass ⋅ time].

**Figure 4.**
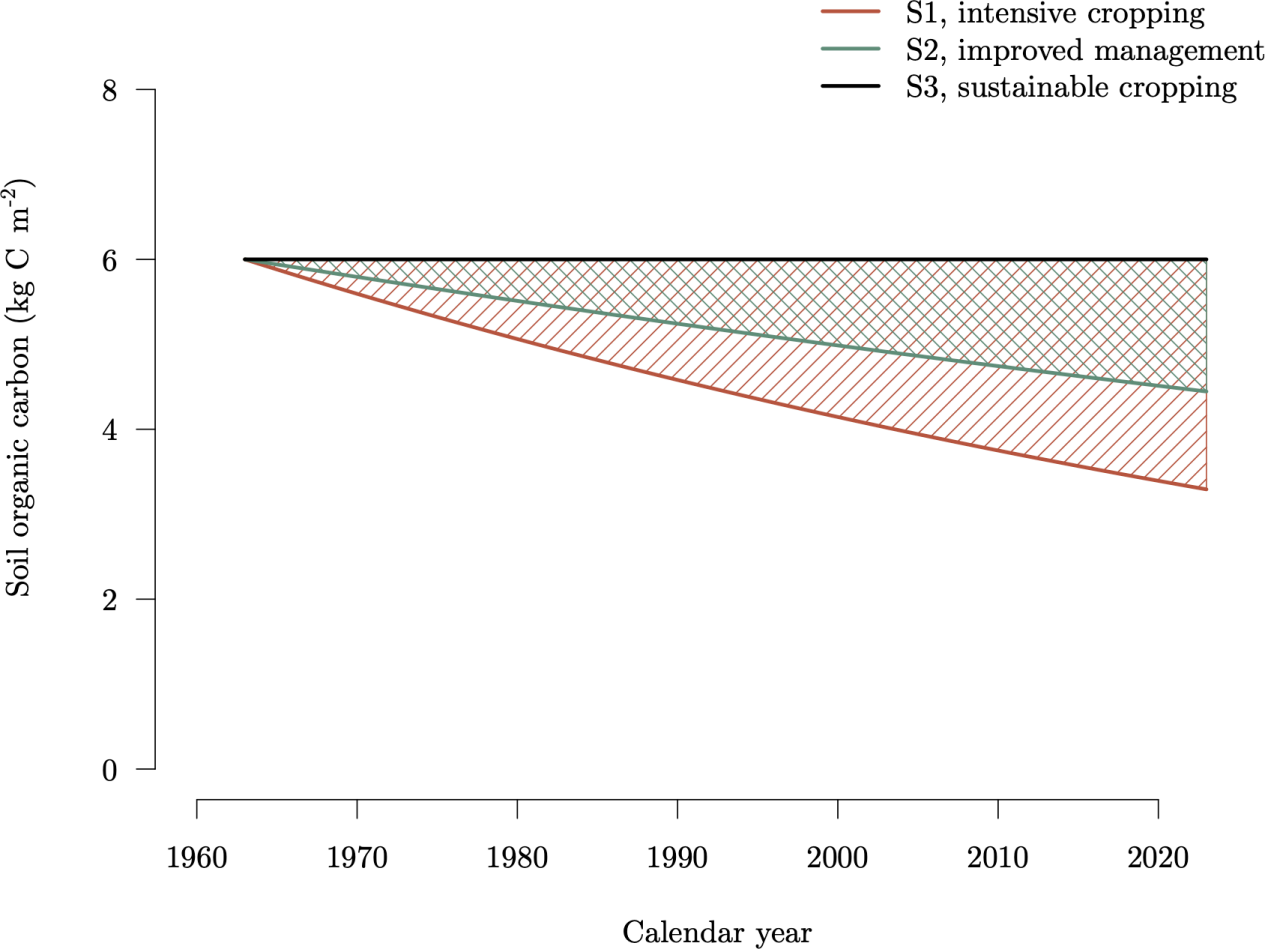
Carbon stocks C (thick lines) and CS (areas marked by oblique lines) for three different cases of soil carbon management. In all three scenarios, $C(t_{0})=6$ kg C m${,}^{-2}$ for $t_{0}=1963$. The time horizon is equal to T=60 years, so $t_{0}+T$ is the calendar year 2023.

To illustrate the importance of comparing on a mass–time basis as opposed to only mass, we look now at a different example. This example is inspired by the trajectories of soil carbon storage at the long-term agricultural experiment in La Estanzuela, Uruguay [38,39]. At this site, soils have been subjected to different forms of cultivation that include S1: intensive continuous cropping system, S2: continuous cropping with fertilization and S3: rotational crop-pasture system. System S1 has led to a continuous degradation of the soils with an important loss of soil carbon during the last 60 years. System S2 has a slower rate of decline than S1, but it still has lost carbon during the 60 years time frame. System S3 does not lose or gain carbon, and it can be interpreted as a sustainable form of crop management [39]. The trajectories of the three systems are mimicked here using exponential functions for the purpose of this example, without explicit use of data from this site (figure 4).

We assume that system S1 with intensive cropping and soil degradation is our baseline, and that we propose to change crop management to reduce the loss of carbon either through S2 or S3. We could claim carbon credits based on a methodology based on stock change only ($\Delta_{b}C$) or on the concept of CS.

Notice that CS for system S3 is zero, no area under the constant black line (figure 4), because this system does not gain or lose carbon during the time frame. For systems S1 and S2, the areas under the curves are in negative values because they express carbon losses with respect to the initial time (table 1).

**Table 1.** Comparison of carbon stocks (C) and carbon sequestration (CS) among three different trajectories of carbon stocks in a cropland soil, with $t_{0}=1963$ and T=60 years. S1: intensive cropping, S2: improved management and S3: sustainable cropping. Values of $\Delta_{t}C$ were obtained using equation (3.4) , $\Delta_{b}C$ with equation (5.1) and $\Delta_{b}$ CS with equation (5.2).

	S1	S2	S3
C (kg m${,}^{-2}$ )	3.29	4.44	6.00
$\Delta_{t}C$ (kg m${,}^{-2}$ )	−2.71	−1.56	0
CS (kg m${,}^{-2}$ yr)	−89.29	−48.98	0
$\Delta_{b}C$ with S1 as baseline (kg m${,}^{-2}$ )	0	1.15	2.71
relative $\Delta_{b}C$ with S1 as baseline (%)	0	35	82
$\Delta_{b}$ CS with S1 as baseline (kg m${,}^{-2}$ yr)	0	40.31	89.29
relative $\Delta_{b}$ CS with S1 as baseline (%)	0	45	100

When accounting based only on carbon stocks, on a time frame from $t_{0}=1963$ with a time horizon of T=60 years, system S2 would avoid the loss of 1.15 kg C m${,}^{-2}$ and system S3 would avoid the loss of 2.71 kg C m${,}^{-2}$ as assessed by the $\Delta_{\text{b}}C$ metric (table 1). This means that system S2 would avoid the loss of 35% of carbon compared with the baseline S1, and system S3 would avoid the loss of 82%. However, these percentages change considerably when assessed with $\Delta_{b}$CS. System S2 avoids 45% of the carbon-year losses, while system S3 avoids 100% of the carbon-year losses (table 1). These percentage differences based on CS are more realistic than when based on C alone; e.g. they show that the more sustainable management system, S3, can avoid 100% of the losses during the entire time frame.

This example is also useful to show that a reference baseline could be defined on an initial time or on the trajectory of a different system. The concept of CS takes into consideration the baseline based on an initial time, while $\Delta_{b}$CS quantifies the effect of both baselines, the initial time and the reference scenario.

## 6. The value of time

Much has been written about the economic and financial aspects of climate change mitigation, how to give value to carbon sequestered over time and how to obtain a net present value based on a discount rate (e.g. [1,2,40–42]). I am not going into detail on that topic here. I only would like to point out that the method of carbon accounting presented here based on the concept of CS facilitates this type of financial analysis because time is explicitly considered in its computation. I also would like to stress that it is important to separate the aspect of *accounting* for time in the physical process of taking up carbon from the atmosphere and retaining it in a reservoir, versus the aspect of *valuing* time. I think these two aspects should be dealt with separately and not in combination as has been advocated previously [1].

The reason why this should be done separately is because the time of carbon retention in an ecosystem is an intrinsic property of the system, determined by physical and biological factors. In ecosystems, the time that it takes carbon atoms to pass through a system, their transit time, depends on the rates of uptake determined by photosynthetic capacity of plants and the environment in which they grow, the allocation of carbon to different plant parts, the rates of plant mortality and transfers of carbon to soils, the activity of the microorganisms that inhabit the soil and other geochemical factors in soil [18,21,43–46]. Some of these processes can be actively managed to increase the time that carbon spends in some of these ecosystem compartments, and it is important that there are incentives, through appropriate accounting methodologies, that can quantify the additional time carbon stays stored in an ecosystem.

A different aspect is to give value to this additional time that carbon can be stored. The valuation of this time does not depend on the physical and biological factors that control the time of carbon storage mentioned above. Valuation of carbon depends mostly on societal, economic and political factors such as the demand for carbon credits, laws that impose limits on emissions, the state of the economy and societal valuation of risk mitigation through CS.

## 7. Why this method is not the same as tonne-year accounting?

The concept of CS presented here only has in common with tonne-year methods [1,2] the form of the unit, [mass ⋅ time], but the concept underlying the computations is completely different.

The methods commonly known as tonne-year accounting are a set of methods that try to make an equivalence between the warming effect of one unit of GHG emission to the atmosphere and the equivalent mass of carbon that should be stored in a system for a period of time [1,2,10]. The resemblance with CS resides in the fact that these tonne-year methods do calculate the integral of an amount of an emission in the atmosphere. They consider the amount of time that an emission of carbon spends in the atmosphere, but they do not consider the time an amount of carbon taken up by a system spends stored [9].

There is an inconsistency in the way tonne-year methods try to relate emissions to sequestration [10,12]. They look at the area under the curve of one single pulse of an emission in the atmosphere and try to provide equivalence of a mass already stored in a natural system (cf. [1]). The concept of CS is independent of carbon dynamics in the atmosphere. It only quantifies the mass and the amount of time of carbon present in a terrestrial system not in the atmosphere.

A more recent version of tonne-year accounting at the global scale has been proposed recently by Matthews *et al*. [6], which is actually more similar to the CS concept described here than the original tonne-year methods. The calculations presented by Matthews *et al*. [6] using an Earth system model showed that integrated carbon storage on land ecosystems is directly proportional to global temperature change with respect to a baseline scenario. Their results suggest a constant proportionality between CS and avoided warming, but our previous theoretical results suggest that this proportionality is not necessarily constant [9].

To relate how a mass of carbon stored over a period of time contributes to avoided warming, a different concept is needed, a concept that quantifies radiative forcing in the atmosphere due to the input–output dynamics of carbon in the atmosphere. I call this concept the climate benefit of sequestration (CBS), which is nothing else than an extension to the concept of absolute global warming potential (AGWP) of a GHG, but in this case taking into consideration that an amount of carbon sequestered by a natural system eventually returns back to the atmosphere. CBS quantifies the radiative forcing that is avoided during the time carbon is sequestered in a natural system, and therefore it is reported in units of W m${,}^{-2}$ yr. It can be compared directly with estimates of AGWP caused by the emission of a greenhouse gas and could serve to determine equivalences between emissions and sequestration in terms of radiative forcing, not in terms of equivalent units of carbon as tonne-year methods attempt. Interested readers can find additional details and equations for the computation of CBS in Sierra *et al*. [9] and Crow & Sierra [22].

## 8. Conclusions

Adopting the conceptual framework provided by compartmental dynamical systems, and integrating the dimension of time in the quantification of carbon removals, can unambiguously solve the issues of quantification of reversals and permanence in the context of carbon accounting.

The concept of compartmental system explicitly distinguishes and quantifies the dynamic nature of carbon input and output fluxes, the carbon contents at any given time and the time it takes new carbon to remain stored (transit time). These concepts can be linked to policy-relevant definitions of carbon removals and reversals, carbon stocks and permanence of carbon removals, as in current discussions on the implementation of the PACM.

In particular, the integration of the time dimension in definitions of CS can help to resolve the problem of permanence by explicitly accounting for the time that carbon remains stored in a particular system. This approach is different from tonne-year accounting methods that attempt to quantify the equivalence of time of carbon in the atmosphere versus a carbon stock. Furthermore, the conceptual approach proposed here could simplify current discussions in the context of the PACM because it would not be necessary to agree on definitions of ‘permanence’ or ‘long-term’ carbon storage. The time dimension would be explicitly incorporated in the quantification of removals and reversals and would be explicitly reported together with the mass of carbon stored in the system (i.e. reporting in [mass ⋅ time]).

Furthermore, integrating the time dimension as proposed here can stimulate the development of project activities that retain carbon already in the system for a long time and keep it out of the atmosphere for as long as possible, which can be particularly useful for projects aimed at the conservation of natural ecosystems.

## Data Availability

Code to reproduce all figures is available at [47].

## Appendix A. Formal definition of a compartmental system

A compartmental dynamical system in the context of the carbon cycle is a set of mathematical functions that track the exchange of carbon with an external environment and the transfers of carbon among internal compartments following mass balance constraints. We denote the mass of carbon stored in a particular compartment i as $C_{i}$, and the total number of compartments as m. The rate of change of the mass in compartment i with respect to time t can be expressed by a differential equation of the form

(A 1)
$$\begin{array}{lcr} & \frac{dC_{i}(t)}{dt}=u_{i}(t)+\sum_{j=1}^{m}f_{ij}(t)-\sum_{j=1}^{m}f_{ji}(t)-r_{i}(t), & \end{array}$$

where the subscript j indicates other compartments in the system. Transfer fluxes among compartments are represented by the functions f, where transfers from other compartments j to compartment i are expressed as $f_{ij}(t)$ and transfers from compartment i to other compartments j as $f_{ji}(t)$. The uptake of carbon from the atmosphere (gross removal flux) into compartment i is expressed by the function $u_{i}(t)$ and release (gross reversal flux) back to the atmosphere as $r_{i}(t)$.

The m differential equations for the entire system can be written in a compact form using vectors and matrices. Furthermore, because the fluxes $f_{ij}$, $f_{ji}$ and $r_{i}$ cannot be larger than the amount of carbon stored in each of the source compartments, these fluxes can be expressed as proportional to the amount of carbon stored in the compartments (see [24], for the mathematical derivation). Therefore, the equation for the entire system can be written as

(A 2)
$$\frac{dC(t)}{dt}=u(t)+\text{B}(t)\cdot C(t),$$

where u(t) is a vector with m components that represent the uptake (gross removals) of each of the compartments at any single time t and C(t) is a vector with m components with the carbon content in each of the compartments at time t. The square matrix B, with m rows and m columns, is a special type of matrix. It is called a *compartmental matrix* because it has three important properties. First, the diagonal elements of the matrix express the rates at which carbon is processed in each compartment. These rates are usually non-positive numbers and are inversely proportional to the time that carbon spends in each compartment. Second, the off-diagonal elements of B are non-negative numbers that express the rates at which carbon is transferred from one compartment to another. If there are no transfers among compartments, the off-diagonal elements would be zero. Third, the sum of the column elements of B cannot be a positive number. This is because, for a mass-balanced system, the carbon that leaves a compartment can only be transferred to other compartments or leave the system entirely.

The total amount of carbon stored in the system at any given time is simply obtained by summing all elements of the vector as C(t)=‖C(t)‖ (vertical bars indicate a sum of all vector elements). Similarly, the total amount of gross removals can be obtained u(t)=‖u(t)‖. The total amount of gross reversals can be obtained by the expression

(A 3)
$$r(t)=-1^{⊤}\cdot \text{B}(t)\cdot C(t),$$

where $-1^{⊤}$ is a transposed vector (rows exchanged with columns) with negative ones. It simply sums all columns of the matrix  B and obtains the proportions of carbon released from each compartment and that are not transferred to other compartments.

At the entire system level, with no disaggregation among compartments, the system can be expressed as

(A 4)
$$\begin{array}{lcr} & \frac{dC(t)}{dt}=n(t)=u(t)-r(t), & \end{array}$$

with no information about what compartments contain carbon and what compartments are the main contributors to removals and reversals.

A robust carbon accounting system would aim at characterizing carbon removals, storage and reversals in the form of equation (A 2). This mathematical representation is very useful for computing a number of system-level metrics such as the transit time of carbon [29], which explicitly quantifies how long the carbon that enters the system remains stored before its final release. However, the representation of the system as in equation (A 2) may be impractical in most cases due to lack of resources for detailed monitoring. An alternative approach would be to monitor all removals and reversals over time as in equation (A 4), or its integration function as in equation (4.3) and then calculate CS as presented in the main text.
